# Usefulness of magnetic resonance imaging-guided vacuum-assisted breast biopsy in Korean women: a pilot study

**DOI:** 10.1186/1477-7819-11-200

**Published:** 2013-08-16

**Authors:** Yeong Yi An, Sung Hun Kim, Bong Joo Kang, Jae Hee Lee

**Affiliations:** 1 Department of Radiology, St. Vincent’s Hospital, College of Medicine, The Catholic University of Korea, 93 Ji-Dong, Paldal-Ku, Suwon, Kyunggi-do 442-723, Republic of Korea; 2 Department of Radiology, Seoul St. Mary’s Hospital, College of Medicine, The Catholic University of Korea, 505 Banpo-Dong, Seocho-Ku, Seoul 137-040, Republic of Korea; 3 Breast Clinic, Human Medical Imaging and Intervention Center, 12-25 Jamwon-Dong, Seocho-Ku, Seoul 137-902, Republic of Korea

## Abstract

**Background:**

Magnetic resonance imaging (MRI)-guided vacuum-assisted biopsy is the technique of choice for lesions that are visible only with breast MRI. The purpose of this study was to report our clinical experience with MRI-guided vacuum-assisted biopsy in Korean women.

**Methods:**

A total of 13 patients with 15 lesions for MRI-guided vacuum-assisted biopsy were prospectively entered into this study between September 2009 and November 2011. Biopsy samples were obtained in a 3-T magnet using a 9-guage MRI-compatible vacuum-assisted biopsy device. We evaluated clinical indications for biopsy, lesion characteristics on prebiopsy MRI, pathologic results, and postbiopsy complication status.

**Results:**

The clinical indications for MRI-guided vacuum-assisted biopsy were as follows: abnormalities in patients with interstitial mammoplasty on screening MRI (*n* = 10); preoperative evaluation of patients with a recently diagnosed cancer (*n* = 3); and suspicious recurrence on follow-up MRI after cancer surgery (*n* = 1) or chemotherapy (*n* = 1). All lesions have morphologic features suspicious or highly suggestive of malignancy by the American College of Radiology Breast Imaging Reporting and Data System category of MRI (C4a = 12, C4b = 2, C5 = 1). In two of the 15 lesions (13.3%, <6 mm), MRI-guided 9-gauge vacuum-assisted breast biopsy was deferred due to nonvisualization of the MRI findings that led to biopsy and the lesions were stable or disappeared on follow up so were considered benign. Of 13 biopsied lesions, pathology revealed four malignancies (4/13, 30.8%; mean size 15.5 mm) and nine benign lesions (9/13, 69.2%; size 14.2 mm). Immediate postprocedural hematoma (mean size 23.5 mm) was observed in eight out of 13 patients (61.5%) and was controlled conservatively.

**Conclusions:**

Our initial experience of MRI-guided vacuum-assisted biopsy showed a success rate of 86.7% and a cancer diagnosis rate of 30.8%, which was quite satisfactory. MRI-guided vacuum-assisted breast biopsy is a safe and effective tool for the workup of suspicious lesions seen on breast MRI alone without major complication. This biopsy may contribute to the early diagnosis of breast cancer in interstitial mammoplasty patients in Korea.

## Background

Magnetic resonance imaging (MRI) of the breast is the most sensitive technique for the detection, diagnosis, and treatment planning of breast cancer with a high sensitivity of 90 to 99%. Although MRI has a high sensitivity in detecting breast lesions, its specificity is comparatively low, ranging from 37 to 72% [1]. Because of the limited specificity of MRI, histopathological verification is required to avoid unnecessary surgical biopsies of benign lesions detected by MRI.

The increasing use of MRI yields a number of MRI-only visible suspicious lesions that cannot be identified by mammography or ultrasonography. A MRI-guided tissue sampling method (MRI-guided percutaneous core biopsy or vacuum-assisted biopsy) is the technique of choice for lesions that are visible only on breast MRI, and it has been performed successfully for many years in the western countries [2-12]. In contrast, second-look ultrasound and ultrasound-guided tissue sampling is usually performed in Korea, and MRI-guided tissue sampling is only performed at a few university hospitals.

Except for the recently published preliminary experience with MRI-guided vacuum-assisted breast biopsy (VABB) in Japan [13-15], there are no published data in Asian or Korean women. The aim of this prospective study was therefore to evaluate our initial clinical experience with MRI-guided VABB of breast lesions visible only on MRI in Korean women. We report our initial clinical experience in 15 cases scheduled for MRI-guided 9-gauge VABB of MRI-detected lesions interpreted as suspicious or highly suggestive of malignancy.

## Methods

### Patient population

This was a prospective study for assessing the usefulness of MRI-guided VABB, approved by the Institutional Review Board of our hospital. Informed consent was obtained from all patients. A total of 13 patients with 15 lesions scheduled for MRI-guided VABB and surgical excision between September 2009 and November 2011 were asked to participate in this prospective study. The patients ranged in age from 35 to 73 years and the median age was 51.4 years.

### Breast MRI technique and lesion characteristics before biopsy

The MRI scans were acquired with the patient in the prone position with a 1.5 T scanner (Achieva; Philips Medical Systems, Best, the Netherlands) equipped with a breast coil.

The MRI images with the Achieva scanner were acquired using the following sequences: sagittal, fat-suppressed, and fast spin-echo T2-weighted imaging sequence (Repetition time/echo time 6,000/100 ms, flip angle 90°, 30 slices, field of view of 320 mm, matrix 424 × 296, number of excitations of 1, 4 mm slice thickness with 0.1 mm interslice gaps, and acquisition time of 2 minutes 56 seconds) and precontrast and postcontrast dynamic axial T1-weighted three-dimensional, fat-suppressed, fat-spoiled gradient-echo sequence (Repetition time/echo time 6.9/3.4, flip angle of12°, 2.0 mm slice thickness with no gap, acquisition time of 1 minute 31 seconds). The images were obtained before and at 0, 91, 182, 273, 364, and 455 seconds after a rapid bolus injection of gadolinium–diethylenetriamine pentaacetic acid (Magnevist; Schering, Berlin, Germany) at 0.1 mmol/kg of body weight.

Two breast radiologists analyzed the imaging findings according to the American College of Radiology Breast Imaging Reporting and Data System categories for MRI [16]. All lesions were classified into category 4 or category 5 on MRI. For all cases, the following lesion characteristics were recorded: type (focus, mass or nonmass enhancement), size, location, and kinetic analysis.

### MRI-guided vacuum-assisted breast biopsy procedure

Biopsies were performed with a 9-gauge MRI-compatible vacuum-assisted biopsy device (Automated Tissue Excision and Collection; Suros Surgical Systems, Indianapolis, IN, U.S). All biopsies were performed by two radiologists specializing in breast imaging (BJK, SHK); all had previous experience in breast MRI and percutaneous biopsies under stereotactic and sonographic guidance. The two radiologists had not previously performed the procedure but had observed or assisted one or more of the experienced radiologists.

The MRI biopsy technique has been previously described in detail [9]. The patient was positioned prone in the 1.5 T magnet (Signa, GE Medical Systems, Milwaukee, WI, USA). The affected breast was placed in a dedicated biopsy compression device using a commercially available grid localizing system. The protocol for MRI-guided VABB at our institution comprised the following six steps: step 1, targeting images (breast compression by the biopsy guidance grid, marking the expected lesion site with a fiducial marker on the skin, and a sagittal T1 fat-saturated three-dimensional spoiled gradient echo image before and after intravenous administration of 20 ml gadodiamide (Omniscan; GE Healthcare, Oslo, Norway) plus 10 ml saline); step 2, determining lesion location and desired depth of probe insertion; step 3, preparing the probe; step 4, placing the device, and performing imaging to confirm the lesion location (sterilization and anesthetization of the breast, placement of the introducer and stylet through the needle guide, removal of the stylet and placement of the obturator inside the introducer, and axial scan to confirm accurate targeting); step 5, performing the biopsy, postexamination images and collecting the specimens (removal of the obturator and insertion of the biopsy device, tissue sampling, and post-biopsy sagittal scan to assess the completeness of tissue acquisition and to examine for the presence of postprocedural complications); and step 6, post-biopsy care of the breast (compression with ice and application of a sterile dressing on the biopsy site).

### Data analysis

Data collection – including clinical indications for breast MRI, patient age, menopausal status, lesion size and type on MRI, biopsy parameters, histologic results of vacuum-assisted biopsy and surgical excision, and complications – was performed by reviewing medical records. The time of the biopsy (minutes) was determined by calculating the interval between the beginning of the MRI localizing sequence and the end of the final MRI sequence acquired. The radiologist performing the biopsy reviewed the MRIs obtained during and after the biopsy to determine the presence and extent of postbiopsy changes, to assess whether the MRI target was sampled or possibly excised.

## Results

### Clinical indications for MRI-guided vacuum-assisted breast biopsy

The clinical indications for MRI-guided VABB were as follows: abnormalities in patients with interstitial mammoplasty on screening MRI (*n* = 10); presurgical evaluation of patients who were recently diagnosed with cancer (*n* = 3); and suspected recurrence on follow-up MRI after cancer surgery (*n* = 1) or chemotherapy (*n* = 1). None of the 15 lesions that were seen on MRI were detected by mammography, diagnostic or second-look ultrasonography.

### Lesion characteristics on pre-biopsy MRI

The MRI-detected lesions included 13 masses and two nonmass enhancements. The mean diameter of lesions on prebiopsy MRI was 1.27 mm (range, 5 to 28 mm). The clinical indications and MRI features with kinetic analysis of 15 lesions are summarized in Table 1. All lesions had morphologic features suspicious or highly suggestive of malignancy according to the American College of Radiology Breast Imaging Reporting and Data System category of MRI (C4a = 12, C4b = 2, C5 = 1).

**Table 1:** Clinical indications, lesion characteristics on prebiopsy magnetic resonance imaging, and pathologic diagnosis in 15 lesions

**Number**	**Indications**	**Lesion characteristics on MRI**					**Pathology**	
		**Type**	**Size (mm)**	**Findings**	**Kinetics**	**BI-RADS category**	**Confirmative methods**	**Pathologic diagnosis**
1	Interstitial mammoplasty, screening	Mass	22	Lobular, smooth, rim	Fast/washout	C4b	VABB/ surgery	IDC
2	Interstitial mammoplasty, screening	Mass	14	Lobular, smooth, homogeneous	Fast/plateau	C4a	VABB/ surgery	IDC
3	Interstitial mammoplasty, ipsilateral breast cancer, chemotherapy follow-up	Mass	8	Irregular, irregular, homogeneous	Fast/washout	C4b	VABB	IDC
4	Interstitial mammoplasty, screening	Mass	18	Irregular, spiculated, heterogeneous	Fast/washout	C5	VABB/ surgery	DCIS
5	Interstitial mammoplasty, screening	Mass	26	Lobular, irregular, heterogeneous	Fast/plateau	C4a	VABB	FA
6	Interstitial mammoplasty, screening	Mass	8	Irregular, irregular, homogeneous	Fast/plateau	C4a	VABB	FA
7	Interstitial mammoplasty, screening	Mass	5	Round, irregular, homogeneous	Fast/plateau	C4a	VABB	FA
8	Interstitial mammoplasty, screening	Mass	28	Lobular, irregular, heterogeneous	Medium/persistent	C4a	VABB	FCC
9	Interstitial mammoplasty, screening	Mass	16	Oval, irregular, heterogeneous	Fast/plateau	C4a	VABB	FCC
10	Interstitial mammoplasty, screening	Mass	6	Oval, irregular, homogeneous	Fast/washout	C4a	VABB	FCC
13	Interstitial mammoplasty, screening	Mass	9	Oval, smooth, heterogeneous	Fast/washout	C4a	VABB	Silicone mastitis
11	Breast cancer, preoperative staging	Mass	8	Oval, smooth, heterogeneous	Medium/plateau	C4a	VABB	FCC
12	Breast cancer follow-up	Nonmass	11	Focal, clumped	Medium/plateau	C4a	VABB	FCC
14	Breast cancer, preoperative staging	Mass	6	Oval, smooth, heterogeneous	Fast/washout	C4a	MRI FU	NA
15	Breast cancer, preoperative staging	Nonmass	5	Segmental, heterogeneous	Medium/plateau	C4a	MRI FU	NA

*BI-RADS* American College of Radiology Breast Imaging Reporting and Data System, *DCIS* ductal carcinoma *in situ*, *FA* fibroadenoma, *FCC* fibrocystic change, *IDC* invasive ductal carcinoma, *MRI* magnetic resonance imaging, *NA* not available, *VABB* vacuum-assisted breast biopsy.

### Biopsy procedure and histopathologic results

MRI-guided VABB was successfully performed in 13 of 15 lesions (86.7%). In two lesions (13.33%), MRI-guided VABB could not be performed because the lesions disappeared on the scheduled day of biopsy. The mean procedural time was 55.7 minutes (range 20 to 90 minutes). An average number of nine specimens were obtained per lesion (range, 6 to 11).

**Figure 1:**
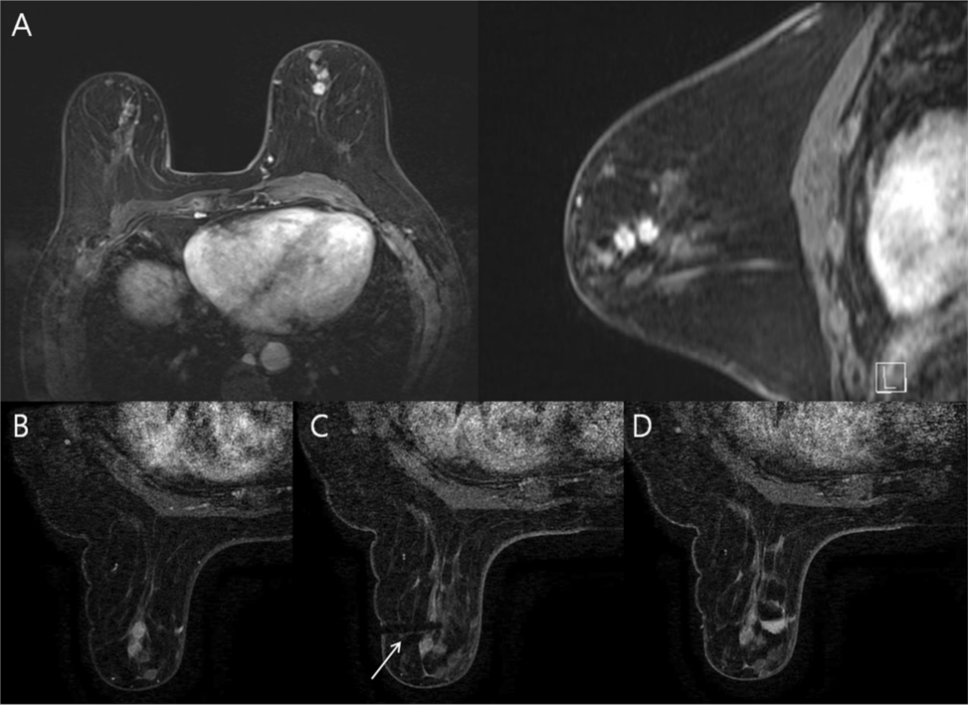
**Example of invasive ductal carcinoma after silicone injection in the breast.** A 73-year-old woman who received silicone injection had a suspicious lesion in the left breast on magnetic resonance imaging (MRI) screening. **(A)** Axial and sagittal contrast enhanced T1-weighted image showing clustered, lobular, rim-enhancing masses with relatively smooth margins in the left breast, which were categorized into American College of Radiology Breast Imaging Reporting and Data System category 4b. **(B)** Axial T1-weighted subtracted dynamic image acquired immediately before biopsy showing clustered enhancing masses. **(C)** Axial T1-weighted subtracted dynamic image of the left breast showing the presence of the obturator (arrow) within the mass. **(D)** Axial T1-weighted subtracted dynamic image of the left breast immediately after 9-gauge MRI-guided vacuum-assisted biopsy showing air and hematoma at the biopsy site. Histologic analysis demonstrated invasive ductal carcinoma.

**Figure 2:**
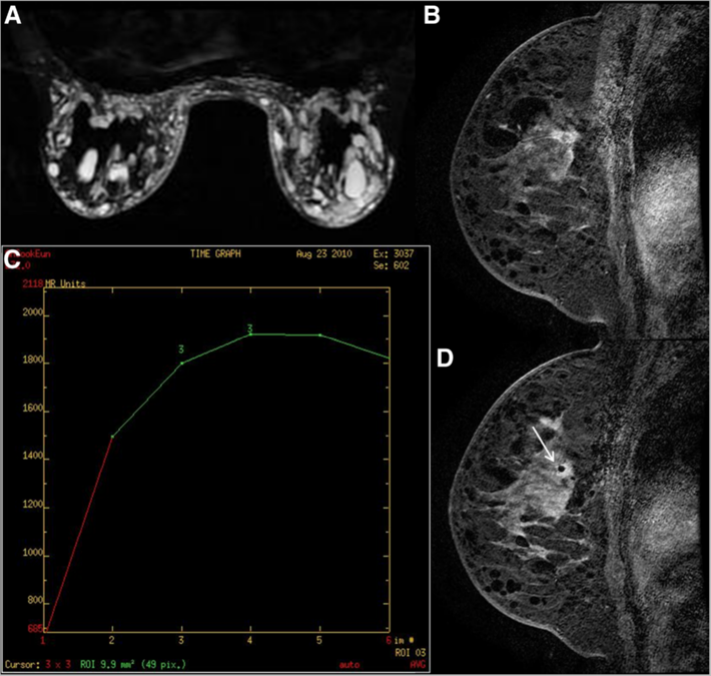
**Example of ductal carcinoma *in situ *after silicone injection in the breast.** A 44-year-old woman who received silicone injection had a suspicious lesion in the left breast on magnetic resonance imaging (MRI) screening. **(A)** Silicone granulomas showing high signal intensities on the inversion recovery MRI with chemically selective water suppression. **(B)** Sagittal T1-weighted subtracted dynamic image of the left breast showing irregular, speculated, heterogeneous enhancing mass, which was categorized into American College of Radiology Breast Imaging Reporting and Data System category 5. **(C)** Dynamic time-intensity curves of the lesion showing an initial fast uptake followed by a delayed washout (type III). **(D)** Sagittal T1-weighted subtracted dynamic image of the left breast showing the obturator tip (arrow) within the mass. Histologic analysis demonstrated ductal carcinoma *in situ*.

**Figure 3:**
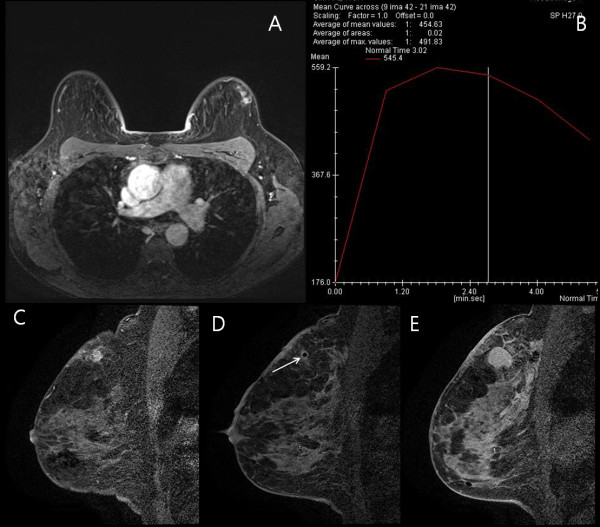
**Example of silicone mastitis after silicone injection in the breast.** A 47-year-old woman who received silicone injection had a suspicious lesion in the left breast on magnetic resonance imaging (MRI) screening. **(A)** Axial contrast enhanced T1-weighted image showing oval, smooth, heterogeneous enhancing mass in the left breast, which was categorized into American College of Radiology Breast Imaging Reporting and Data System category 4a. **(B)** Dynamic time-intensity curves of the lesion showing an initial fast uptake followed by a fast washout in the delayed phase (type III). **(C)** Sagittal, T1-weighted subtracted dynamic image acquired immediately before biopsy showing heterogeneously enhancing mass in the 12:00 hours position. **(D)** Sagittal T1-weighted subtracted dynamic image acquired after placement of the obturator showing low signal within the lesion (arrow), indicating the location of the obturator tip. **(E)** Sagittal T1-weighted subtracted dynamic image immediately after completion of tissue acquisition showing hematoma formation at the biopsy site. Histologic analysis demonstrated silicone mastitis.

Histopathological results of the VABB and findings of the surgical specimens are summarized in Table 1. Of the 13 lesions sampled, four lesions were malignant (30.8%, 4/13) (Figure 1 and Figure 2) and the remaining nine lesions were benign (69.2%, 9/13) (Figure 3).

Among the cases of confirmed malignancies, three lesions were invasive ductal carcinoma (Figure 1) and one was ductal carcinoma *in situ* (Figure 2). All cancers diagnosed by VABB were the mass type of enhancement. Malignant lesions diagnosed by VABB were surgically excised in three out of four cases. One patient did not undergo surgery due to bone metastasis detected by bone scan and positron emission tomography–computed tomography. Residual cancer was found at surgery in two out of three patients. One patient, in whom the MRI target was considered to have been completely excised, had no residual lesion. All benign diagnoses were confirmed by imaging–histologic correlation and by follow-up MRI at 6 to 12 months after biopsy. There were no missed cancers in the patients having benign lesions with concordant histology on MRI-guided biopsy.

### Lesions with deferred biopsy

**Figure 4:**
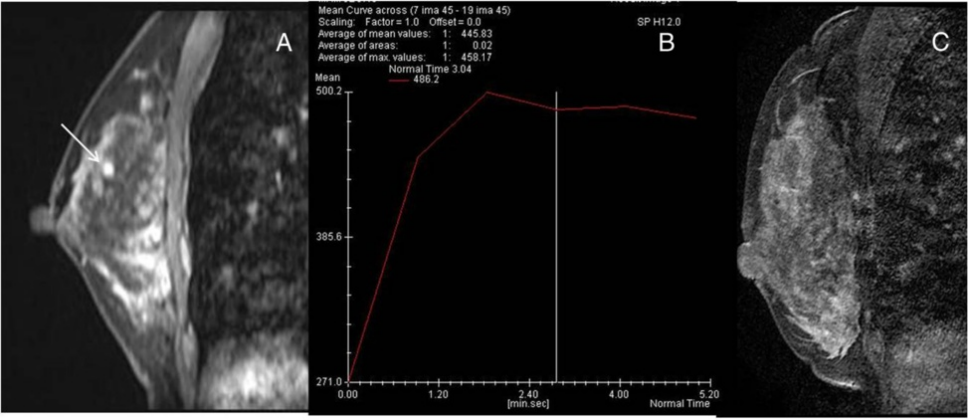
**A 38-year-old woman with breast cancer on preoperative magnetic resonance imaging for staging. (A)** Sagittal, contrast enhanced T1-weighted image showing oval, smooth, heterogeneous enhancing mass in the right breast, measuring 6 mm, which was categorized into American College of Radiology Breast Imaging Reporting and Data System (BI-RADS) category 4a. **(B)** Dynamic time-intensity curves of the lesion showing an initial fast uptake followed by a fast washout in the delayed phase (type III), which was categorized into BI-RADS category 4a. **(C)** Sagittal, contrast enhanced T1-weighted image on the day of biopsy showing no suspicious lesions, and hence magnetic resonance imaging-guided breast biopsy was cancelled.

There were two lesions in which the biopsy was deferred. The lesion type was foci measuring 4 mm and a small mass measuring 6 mm on the initial MRI (Figure 4). Follow-up MRI within 6 months revealed resolution or decrease in the size of the lesions.

### Postbiopsy complications

MRI performed immediately after biopsy showed that complete removal of the target lesion was achieved in one patient (7.7%), partial removal of the target lesion was achieved in seven patients (53.8%), and there was difficulty in assessing the removal of the target lesion due to bleeding in five patients (38.5%).

Postprocedural hematoma formation was observed in eight patients (61.5%), and was controlled conservatively. The mean size of the hematoma was 23.5 mm (range, 21 to 28 mm). Postprocedural defect was observed in three patients and the mean size of the defect was 14.3 mm (range, 11 to 18 mm). Other complications (significant bleeding, infection, vasovagal reaction, contralateral skin piercing) were not observed.

## Discussion

This study reports our early experience with MRI-guided VABB. We had no prior experience in MRI-guided VABB. Our initial experience showed that the success rate of MRI-guided VABB was 86.7% (13/15). This rate is slightly lower than the 96 to 100% technical success rates reported in prior studies of MRI-guided VABB [6-9,11,12,14]. The median procedural time in our study was 55.7 minutes. The median time of MRI-guided VABB with the same device in prior studies ranged from 30 to 38 minutes. MRI-guided VABB revealed cancer in four of 13 cases (30.8%), similar to prior clinical reports of this method. Potential failure of MRI-guided VABB were mainly due to the nonvisualization of lesions on the day of biopsy, which may have been caused by changes in the hormonal status or probably due to excessively strong compression of the breast during biopsy. On gaining experience, MRI-guided VABB can be performed in an even shorter amount of time and can have a higher technical success rate than that reported in this study.

Compared with previous reports of studies on MRI-guided VABB, our study included a higher proportion of interstitial mammoplasty cases (11 out of 15, 73.3%), which has not been reported previously (Figure 1, Figure 2 and Figure 3). The malignancy rate in 11 interstitial mammoplasty cases was 36.4% (4/11), and the mean size of detected cancer was 1.5 mm (range, 8 to 22 mm). Patients who had received silicone or paraffin injection could not be easily or properly evaluated by palpation, mammography, or sonography. As a consequence, breast cancers in patients with interstitial mammoplasty were detected at an advanced stage. Dynamic breast MRI is the modality of choice to detect breast cancer, and MRI-guided biopsy is the only method that can confirm suspicious enhancing breast lesions in these patients. According to our previous study [17], the mean diameter of the detected cancers in these patients was 5.5 cm (range 4.8 to 7.1 cm). In this study, cancers diagnosed by MRI-guided VABB were smaller in size and at an earlier stage compared with those in our previous publication.

The present study has some limitations. The main limitation of this study was the small number of cases. Another limitation was the short period of follow-up in some cases (14 to 35 months) with benign lesions. Thirdly, we did not correlate the hormonal status of the patient and the lesion visibility on MRI. A better approach would be to obtain the patient’s menstrual history and usage of hormone replacement therapy before MRI examination.

## Conclusions

MRI-guided VABB is a safe, efficient and effective tool without any major complications in Korean women. During our initial experience a success rate of 86.7% was achieved, which was quite satisfactory. MRI-guided VABB may contribute to the early diagnosis of breast cancer in interstitial mammoplasty patients in Korea.

## Abbreviations

MRI: Magnetic resonance imaging; VABB: Vacuum-assisted breast biopsy.

## Competing interests

The authors declare that they have no competing interests.

## Authors’ contributions

YYA and BJK participated in conception of the study, data collection and analysis, and drafted the manuscript. SHK and JHL participated in data collection and analysis. All authors read and approved the final manuscript.
